# Dynamic Stability of Coral Reefs on the West Australian Coast

**DOI:** 10.1371/journal.pone.0069863

**Published:** 2013-07-29

**Authors:** Conrad W. Speed, Russ C. Babcock, Kevin P. Bancroft, Lynnath E. Beckley, Lynda M. Bellchambers, Martial Depczynski, Stuart N. Field, Kim J. Friedman, James P. Gilmour, Jean-Paul A. Hobbs, Halina T. Kobryn, James A. Y. Moore, Christopher D. Nutt, George Shedrawi, Damian P. Thomson, Shaun K. Wilson

**Affiliations:** 1 Science Division, Department of Environment and Conservation, Marine Science Program, Kensington, Western Australia, Australia; 2 Commonwealth Scientific and Industrial Research Organisation, Marine and Atmospheric Research, Wembley, Western Australia, Australia; 3 School of Veterinary and Life Sciences, Murdoch University, Murdoch, Western Australia, Australia; 4 Biodiversity and Biosecurity Branch, Department of Fisheries Western Australia, Hillarys, Western Australia, Australia; 5 Australian Institute of Marine Science, The UWA Oceans Institute, Crawley, Western Australia, Australia; 6 The UWA Oceans Institute, Crawley, Western Australia, Australia; 7 The School of Plant Biology, University of Western Australia, Crawley, Australia; University of Vigo, Spain

## Abstract

Monitoring changes in coral cover and composition through space and time can provide insights to reef health and assist the focus of management and conservation efforts. We used a meta-analytical approach to assess coral cover data across latitudes 10–35°S along the west Australian coast, including 25 years of data from the Ningaloo region. Current estimates of coral cover ranged between 3 and 44% in coral habitats. Coral communities in the northern regions were dominated by corals from the families Acroporidae and Poritidae, which became less common at higher latitudes. At Ningaloo Reef coral cover has remained relatively stable through time (∼28%), although north-eastern and southern areas have experienced significant declines in overall cover. These declines are likely related to periodic disturbances such as cyclones and thermal anomalies, which were particularly noticeable around 1998/1999 and 2010/2011. Linear mixed effects models (LME) suggest latitude explains 10% of the deviance in coral cover through time at Ningaloo. Acroporidae has decreased in abundance relative to other common families at Ningaloo in the south, which might be related to persistence of more thermally and mechanically tolerant families. We identify regions where quantitative time-series data on coral cover and composition are lacking, particularly in north-western Australia. Standardising routine monitoring methods used by management and research agencies at these, and other locations, would allow a more robust assessment of coral condition and a better basis for conservation of coral reefs.

## Introduction

Effective management and conservation of any natural resource relies on an assessment of its current status and how it changes spatially and temporally. A common approach to monitoring the health of natural resources is to adopt an indicator that will respond to disturbance, establish baselines and assess changes through time, all of which provide an impetus for management action. On coral reefs, commonly used indicators of health are: coral cover [1], [2], [3], [4], reef fish or shark abundance [5], herbivory [6], coral recruitment [7], or a combination of multiple resources [8], [9], [10]. Establishing a baseline is essential for monitoring programs [11], particularly in areas where impacts have already occurred and caused a shift in communities [12]. However, long-term (multi-decadal) time-series datasets, which allow meaningful ecosystem-wide assessments, rarely exist over wide spatial scales (100 s km). One method that has been used to overcome sporadic and disconnected data collection is meta-analysis [1], [2], [13], [14], [15]. This approach allows the construction of datasets over a wide spatial and temporal range whilst assessing congruence of patterns discerned from different studies.

Hard coral cover is the most widely used indicator of coral reef condition, as skeletal accretion by scleractinian corals forms the basis of all tropical coral reefs and provides habitat and food resources for a range of vertebrate and invertebrate fauna. Several broad-scale studies have documented a rapid decline in coral cover [13], [14], [16], as well as studies focusing on the Indo-Pacific region [e.g., 1,2,4,17]. Large-scale decline in coral cover can be related to both natural events (e.g., storms, disease, recruitment failure), and human activities that induce overexploitation of resources, degraded water quality, and climate change [11], [18], [19].

Changes in coral cover can document reef decline or recovery over large temporal and spatial scales [3], [17], although, if used alone, it can mask shifts in community composition [11]. In addition to global declines in coral cover, community composition also appears to be changing due to taxonomic differences in their susceptibilities to the frequency and severity of impacts [20], [21]. In some instances, impacts have caused large shifts away from the typical Acroporidae-dominated community composition [22], [23]. Shifts in community composition could potentially have profound effects on associated fish [24], [25] and invertebrate species [26] that rely on specific coral species or morphologies (i.e., branching, massive, tabulate). Considerable reductions in Acroporidae assemblages are well known in the Caribbean [27], and there are also examples of similar community shifts in coral cover in the Indian Ocean [10], [28], [29].

Research in the Indian Ocean has mainly focused on the western and central areas where changes in live coral cover and community composition have been attributed to coral bleaching [2], [30], [31]. However, relatively little research has been published on the community composition and coral cover on reefs in the South Eastern Indian Ocean [32], [33]. The unique southward flowing Leeuwin Current dominates the oceanography of the West Australian (WA) coastline [34], providing suitable hydrographical conditions for extensive coral reef growth [35]. Moreover, this coastline is an area of high endemicity [36] and includes two World Heritage Areas, Ningaloo Reef and Shark Bay.

Diversity of coral communities on the south-eastern seaboard of the Indian Ocean has been described [37], [38], [39], [40], however, there has been no synthesis of quantitative data that examines cover and composition on a broad scale. Much of the available information focuses on Ningaloo Reef due to the uniqueness and extent of this reef system [39], a recent surge in targeted State Government funding, as well as its accessibility and exposure to tourism [41], , which has generated the most detailed and spatially-explicit data sets on coral cover in WA. Understanding long-term trends in coral communities at Ningaloo and assessing spatial variance along the coast are essential if we are to assess the effects of local-scale disturbances and management relative to global issues such as climate change.

Increasing industrial and residential development pressure on the north-west coast of Australia, as well as recent extreme climate events [e.g., 43,44,45,46,47] have increased the urgency to obtain synoptic and historical perspectives on coral cover in this region. In our study, we examined spatial coral cover for the west Australian coast and offshore territories, which make up much of the south eastern seaboard of the Indian Ocean, and focused our temporal component on Ningaloo Reef. Specifically, we aimed to; i) describe the spatial distribution in coral cover and composition for West Australia and offshore territories to determine reef condition; ii) identify gaps in knowledge of coral reef areas to help direct future research; iii) determine long-term (25 yr) spatial and temporal patterns in coral communities at Ningaloo Reef; and iv) assess environmental factors impacting on coral cover at Ningaloo, which provides an indication of reef stability and condition through time.

## Materials and Methods

### Data Sourcing & Filtering

**Figure 1:**
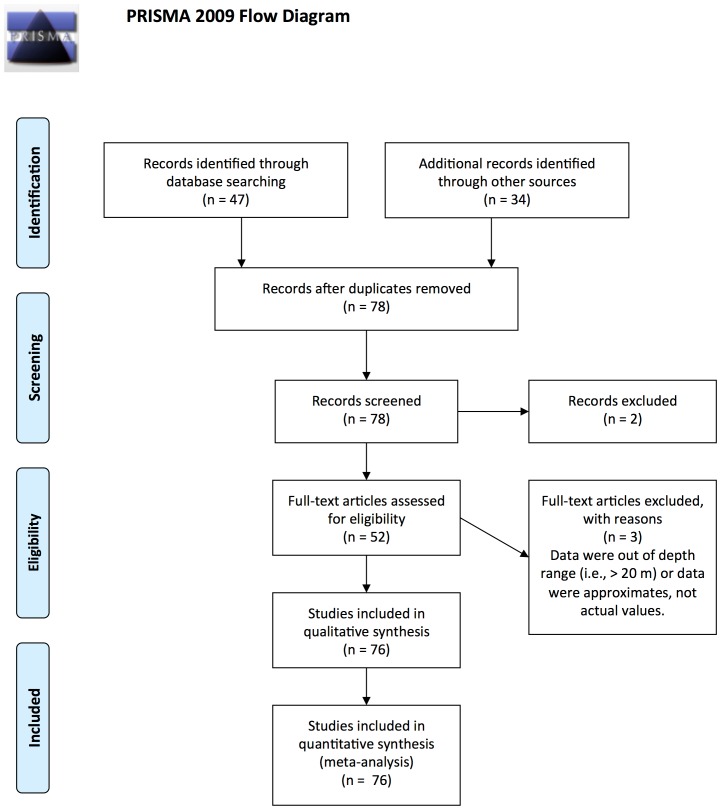
Literature identification and filtering for inclusion in meta-analysis of coral cover patterns in Western Australia.

There were three data sources considered for inclusion in our study: 1) peer-reviewed literature (journal articles), 2) grey literature (reports), and 3) unpublished data that were collected for research purposes or environmental monitoring programs (Fig. 1). Studies that provided an estimate of hard coral cover and composition from sites off the coast of WA were included in our database. Studies were included in our database provided they also included other metadata including depth (or depth range), habitat type(s) surveyed, and collection method. Data collected from *in-situ* monitoring were used, as opposed to data gathered from air-borne or satellite remote sensing to avoid error associated with differences in spatial resolution. Data collection methods included still photography, video and *in-situ* visual estimates from transects, quadrats, manta-tows, towed video, and Autonomous Underwater Vehicles. Although there are no studies that assess compatibility of coral community estimates from all these techniques, comparisons of two or more of these methods indicate estimates of percent coral cover and broad functional/taxonomic groups are comparable [13], [56], [57], [58]. We were primarily interested in coral cover of shallow coastal and offshore areas due to availability of data, and increased vulnerability to prominent natural and anthropogenic impacts [59]. We therefore restricted data collection to surveys that were done at depths ≤20 m, with the majority of data collected at average depths of ≤10 m (∼80% of surveys). This also reduced potentially confounding results of coral cover patterns due to varying depth gradients. Surveys included were also generally restricted to subtidal zones, although intertidal exceptions were in the Kimberley region, where tidal amplitudes can be up to 10 m [60]. A complete list of studies and sources from which data were derived is included in Table S1.

For assessing the impacts of disturbances on coral cover at Ningaloo Reef, cyclone frequency and intensity information were acquired from the Australian Bureau of Meteorology (BOM) http://www.bom.gov.au/cyclone/history/tracks/index.shtml. Hourly data taken from when cyclones where within a 200 km radius of Ningaloo Reef (Coral Bay –used as a center point) were used to estimate average intensity and frequency of cyclones in the region from 1987 to 2010. Cyclone intensity is measured from the center of the cyclone and recorded in hectopascals (hPa).

Water temperature was derived by applying a linear model to compare sea surface (SST) temperature from the NOAA Virtual Station at Ningaloo (http://coralreefwatch.noaa.gov/satellite/vs/index.html?lat=-20&lng=128&zoom_level=4) with *in-situ* water temperature measured at Point Murat. The model was then used to adjust historic temperature estimates derived by satellite (Baldock et al. unpublished data). We considered this to be an important correction of historical satellite-derived SST estimates, as previous estimates from Western Australia have often been shown to be erroneous by up to 2°C [61], [62].

### Study Area

**Figure 2:**
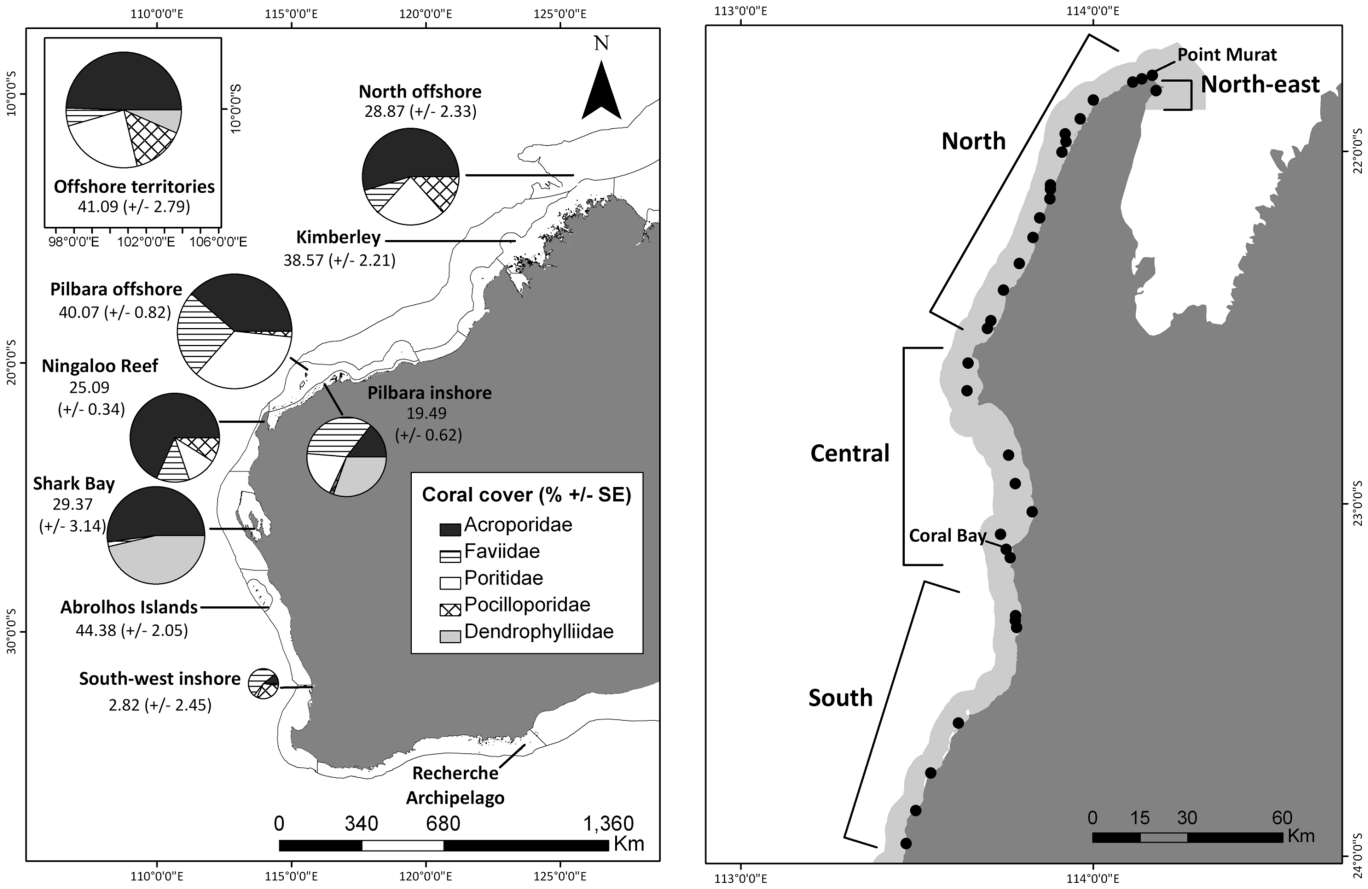
Map of study Western Australian coral monitoring sites showing: A) Dominant coral family composition for each sampling region and absolute coral cover (% ± SE), and B) Ningaloo Reef with survey sites (), where light grey shadow area denotes Ningaloo Marine Park boundary. The size of pie charts is relative to overall percentage of total absolute coral cover. Pie charts represent contribution of dominant coral families relative to one another for each region. Lines connect pie charts with corresponding regions. Coral cover and composition for regions within Western Australia based on the last 10 years of data collected.

The 12,500 km coastline of WA spans latitudes from 13° 30′ S to 35° 08′ S (Offshore territories 12° - 10° S) (Fig. 2a), and includes a diverse range of coral habitats such as fringing reefs, coral atolls, and reef plateaux [37]. Many of the coral reef systems along the WA coast are influenced by the Leeuwin Current, which is a southward flowing, narrow (<200 km) boundary current of warm water with low salinity and low nutrients [34]. The coastal and marine environments in Australia are currently classified into bioregions under the Integrated Marine and Coastal Regionalisation of Australia (IMCRA), which have been established from ecological information for management and planning purposes [48].

Information from coral surveys along the entire west Australian coast, and associated territories, were considered for analyses, with a more detailed focus directed on Ningaloo Reef (21° 51′S, 114° 10′E - 23° 34′S, 113° 43′E) due to the extent of unpublished data on coral communities from this reef system, in addition to several recently published sources of information [e.g., 49,50,51]. Ningaloo is Australia’s largest fringing reef (∼300 km) and the Ningaloo Marine Park, managed by the Department of Environment and Conservation (DEC), covers 263,343 ha of reef and associated habitats [52]. There are at least 217 species of hermatypic corals from 54 genera identified from Ningaloo Reef [37], of which the dominant family is Acroporidae [50].

Four distinct sub-regions occur along Ningaloo Reef, which differ considerably in their geomorphology (Fig. 2b). The North-east sub-region is the Bundegi Reef complex (21° 49′S, 114° 10′E), which is situated in the Exmouth Gulf and is a turbid low energy reef that is protected from the prevailing south-westerly winds and high wave energy to which the west side of the reef is exposed [53]. The North sub-region begins at Vlamingh Head (21° 48′S, 114° 6′E) and runs south to Point Edgar (22° 21′S, 113° 23′E). This sub-region is located near the shelf break and, unlike the other sub-regions, the 200 m depth contour is only 10 km offshore [54]. The Central sub-region begins at Point Cloates (22° 25′S, 113° 24′E) and spans to Monck Head (23° 6′S, 113° 27′E), and is uniquely characterised by both the increased width of its lagoon and a distinctive recirculation of minor currents and eddies around Point Cloates [55]. Beginning at Pelican Point (23° 19′S, 113° 46′E) and extending southwards to the end of the Ningaloo Marine Park at Red Bluff (24° 3′S, 113° 24′E), the South sub-region is differentiated by the absence of a lagoon where the reef flat extends from the reef crest to the beach and a broader, shallower offshore coastal shelf than the central and northern regions.

### Data Analysis

#### West Australian coast and offshore territories

We organised survey data into nine regions that were based on the Integrated Marine and Coastal Regionalisation of Australia (IMCRA) bioregions (Commonwealth of Australia 2006) (Fig. 2a). Some regions were combined due to paucity of data and their similarity of coral community composition and known distributions [37]. We combined Central West Coast (CWC) and Leeuwin Naturaliste (LNE) bioregions, which we have labeled ‘South-west inshore’, the North-west Shelf (NWS) and Oceanic Shoals (OSS) bioregions, which we have labeled ‘North offshore’, and Australian Offshore Territories at Christmas Island and the Cocos (Keeling) Islands, which we have called ‘Offshore territories’.

Only studies that surveyed areas of hard substrata that were suitable for coral growth and development were included in our analysis. Studies were also required to have area (m^2^) sampled, which allowed us to weight studies. Where only transect length was provided, (e.g., line intercept transects), we assumed a 1 m width to get an approximation of area covered. We calculated transect area by multiplying transect length by width, which standardised area estimates across surveys. To provide a current estimate of coral cover, we only used the past 10 years of data collected for each region. We estimated average coral cover per region using weighted arithmetic means [63], where area sampled (m^2^) was used to provide weightings for each study:
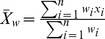
(1)where *w_i_* is the area sampled and *x_i_* is coral cover. To calculate standard errors around each mean estimate, we used a bootstrap sampling with replacement [64]. Using weighted means places a greater emphasis on studies that have sampled larger areas, and has been used in previous meta-analyses to account for differences in effort among studies [65], [66].

As not all studies collected coral community composition data, a subset of these studies (∼56%) was used to estimate community composition of dominant families for each region (Acroporidae, Faviidae, Pocilloporidae, Poritidae, and Dendrophylliidae). We did not attempt a temporal comparison of either coral cover or coral composition along the entire western coast of Australia due to sporadic and unbalanced sampling effort for most regions.

#### Ningaloo Reef

Ningaloo Reef was the most suitable region for assessing spatial and temporal dynamics of coral cover and composition due to the presence of established monitoring sites, which have been repeatedly surveyed. Surveys span a 25-year period between 1987 and 2012. We grouped data from Ningaloo into four sub-regions (North-east, North, Central, and South, Fig. 2b) because of their well-documented relevance to hydrographic and bio-geographical features [e.g., 50,54,55].

We estimated average coral cover per year for Ningaloo as a whole and for each sub-region using weighted arithmetic means [63], where area sampled (m^2^) was used to provide weighting for each study (equation 1). A subset (∼47%) of the Ningaloo data was suitable for assessing patterns in community composition. Average annual percent coral cover was calculated for four common families of corals (Acroporidae, Faviidae, Pocilloporidae, and Poritidae). Due to the reduced size of the data set, data were grouped into time periods of 5-year spans between 1990 and 2012, the last time period (2010–2012) consisting of only 3 years. Five-year spans were determined to have sufficient temporal resolution to establish major changes to total coral community composition [e.g., 3].

We used a square root transformation to ensure data fitted a Gaussian distribution and then applied linear mixed effects models (LME) [67] to assess the strength of the relationship between coral cover (unweighted estimates – full dataset) through time at Ningaloo with latitude, habitat type, and size of area surveyed. A mixed model approach was adopted as it allowed us to explore the potential influences of multiple environmental variables, while including sampling site as a random effect. Non-independence of data (i.e., spatial and temporal autocorrelation) is a potential source of error in meta-analytical studies, however, identifying which data are auto-correlated from published studies is generally impossible, as raw data are rarely available [15]. We tested for autocorrelation in our working dataset of summarised values, and found that temporal auto-correlation was present (Figure S1). A mixed-model approach allowed us to include a correlation structure to account for temporal non-independence. Specific depth values at the site level were often not provided in studies we reviewed (rather a depth range), which precluded the inclusion of this variable in models. We were also unable to include cyclone intensity and water temperature as explanatory variables due to lack of spatial resolution in the datasets. We classified habitat types as ‘forereef’ (includes reef slope, ∼8.6% of samples), ‘backreef’ (∼75%), ‘lagoon’ (includes patch reefs, ∼9%), and ‘reef flat’ (∼6.8%).

Temporal trends in coral cover (yearly weighted averages) at Ningaloo Reef (all sub-regions combined) and for each sub-region independently were examined using linear and non-linear models (4^th^ order polynomial and natural cubic splines). We selected these models because of uncertainty whether coral cover followed linear or non-linear trends. We ranked and weighted all models using Akaike’s Information Criterion corrected for small sample sizes (AIC*_c_*), associated weights (*w*AIC*_c_*), and percent deviance explained (%DE) [68]. All analyses were carried out in program R [69] using packages ‘plyr’, ‘boot’, and ‘nlme’.

## Results

### West Australian Coast and Offshore Territories

Data from 121 surveys were obtained between 1980 and 2012. These surveys represent data collected in 76 separate studies, of which 35 (46%) were done at Ningaloo Reef (Table 1 and Table S1). Both Ningaloo Reef and North offshore regions had the largest areas surveyed (>250, 000 m^2^ each), while Shark Bay had the lowest (1,800 m^2^).

**Table 1:** Summary of data sourced for meta-analysis in our study.

WA region	Survey period	Number ofsurveys	Area surveyed (m^2^)	Journal articles	Reports	Unpublisheddata	Total studies
*North offshore*	1993–2010	32	273,985	3	9	1	13
*Offshore territories*	2008–2012	4	13,500			1	1
*Kimberley*	2007–2010	3	3,172	1	2		3
*Pilbara offshore*	2006–2011	4	16,250		1	1	2
*Pilbara inshore*	1985–2009	11	15,315		10		10
*Ningaloo Reef*	1980–2012	45	258,345	5	16	14	35
*Shark Bay*	1996–2011	4	1,800		1	1	2
*Abrolhos*	2004–2011	5	11,100	1	1	1	3
*South-west inshore*	1999–2011	13	54,145	1	3	3	7
**Total**		**121**	**643,742**	**11**	**43**	**22**	**76**

Note: Area surveyed might be inflated in regions where repeat surveys were done over time at fixed sampling sites.

The four regions with the highest overall mean coral cover were the Abrolhos Islands, Offshore territories, Pilbara offshore, and the Kimberley (>38% cover) (Fig. 2a). Corals from the family Acroporidae were prominent in most regions, and accounted for 3–40% of corals present (Fig. 2a). The greatest contribution of Acroporidae was found in communities from the most northern extent of our study area, the Offshore Territories (19.2% ±1.5 SE), and the lowest in the most southern part of our study area around South-west inshore (1.1% ±0.4 SE). Acoporidae was also quite common within Shark Bay (15.1% ±3.5 SE). Poritidae showed a similar pattern to Acroporidae in that there was a higher relative contribution in the North offshore region (9.4% ±0.7 SE) than the South-west inshore region (0.3% ±0.2 SE). There was some evidence to suggest that both Acroporidae and Poritidae were less abundant in cover with increasing latitude, albeit non-significant relationships (Adj. R^2^ = 0.42, p = 0.067 and Adj. R^2^ = 0.45, p = 0.057, respectively) (Table S2). Faviidae was found throughout all regions, ranging from 0.5% (±0.1 SE) in Shark Bay to 17.5% (±0.5 SE) at Pilbara inshore. Corals from the family Dendrophyllidae were present throughout most of the regions, although they were most abundant within the inshore in the Pilbara (15.7% ±0.5 SE) and in the more sheltered embayments of the Shark Bay region (13.4% ±0.5 SE).

Despite high coral cover, gaps in coral family level data were identified for the Abrolhos Islands and the Kimberley. Indeed, the Kimberly had the least surveys conducted (*n* = 3), which was followed by Offshore territories, Pilbara offshore and inshore, and Shark Bay (*n* = 4 each) (Table 1). No quantitative data were available on coral cover or community composition in the far south-east of West Australia, in the Recherche Archipelago.

### Ningaloo Reef

**Figure 3:**
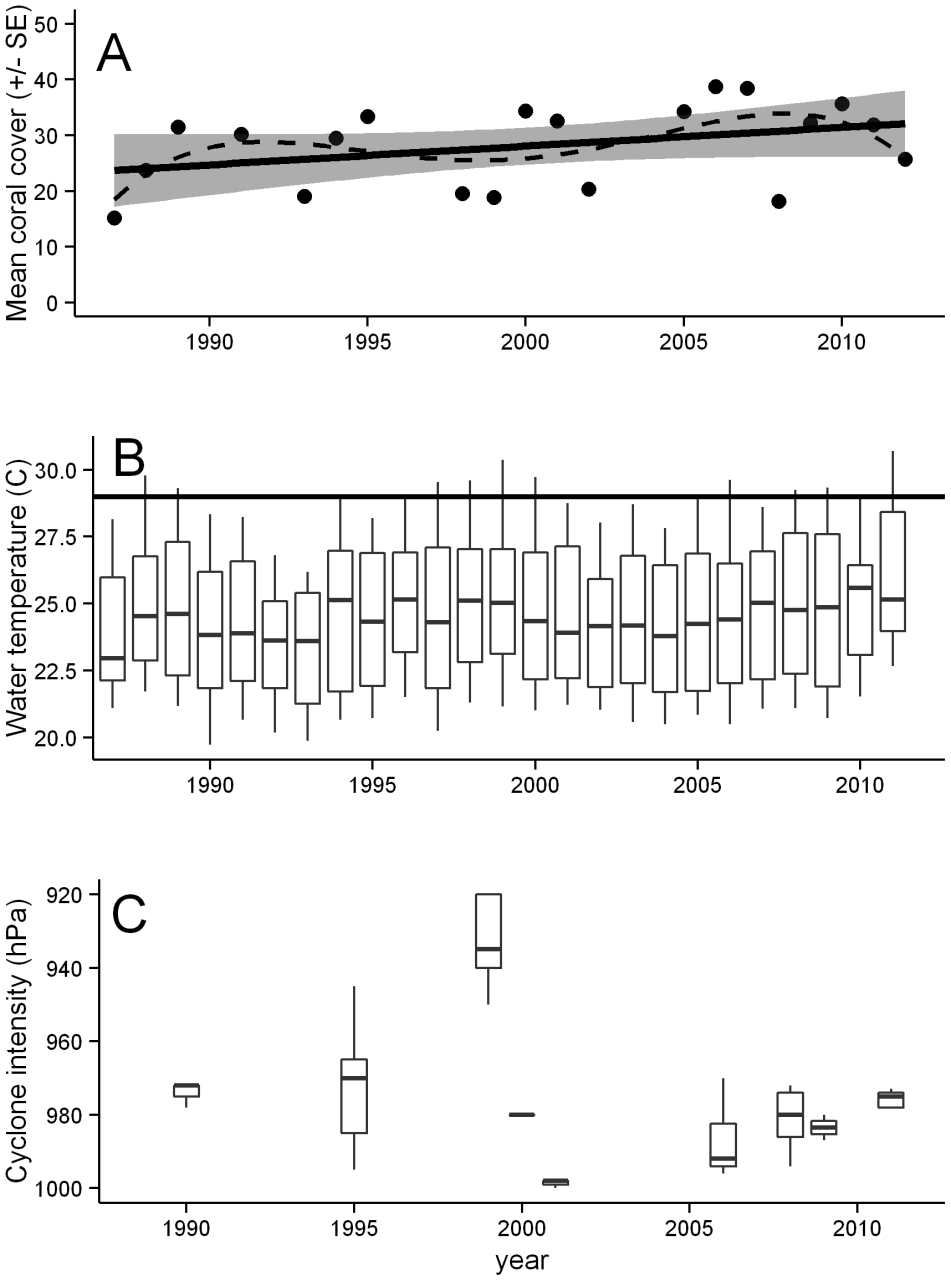
A) Coral cover at Ningaloo Reef between 1987 and 2010. Lines fitted using a linear regression (solid line with standard errors) and a 4^th^ order polynomial regression (dashed line. B) Water temperature from Point Murat, at the northern end of Ningaloo Reef – thick black line represents approximate bleaching threshold for corals based on NOAA Coral Watch http://coralreefwatch.noaa.gov/satellite/vs/australia.html#Ningaloo_Australia. C) Cyclone frequency and intensity measured in Hectopascals at Ningaloo Reef. Each box represents central pressure measured for individual cyclones, except for 1995 (values averaged for two cyclones). Upper and lower whiskers in B and C represent the 1^st^ and 3^rd^ quartiles.

Total average coral cover across Ningaloo Reef for the entire 25 year period (1987–2012) was 28.1% (±2.1 SE). While there was variation in coral cover through time with cover dropping below 20% during certain periods, overall coral cover has remained stable (Fig. 3a). A linear model provided the best fit for Ningaloo overall (*_w_*AIC*_c_* = 0.45), although this model did not differ to the intercept only model (null model), suggesting no strong linear increases or declines between 1987 and 2012 (Table 2). Overall variation in coral cover at Ningaloo was best explained by latitude (10%) with comparatively little variation explained by habitat type, or survey area (Table 3). The period with consecutive years where coral cover was ≤20% was around 1998/1999, which coincided with both a peak in average water temperatures (Fig. 3b) and a severe cyclone (Fig. 3c).

**Table 2:** Model ranking results of relationship between coral cover over time for sub-regions at Ningaloo Reef.

Subregion	Model	*df*	-LL	AIC_c_	_w_AIC_c_	%DE
All subregions combined	Intercept only (cover ∼1)	18	−67.8347	139.6694	0.3118	0.0000
	Spline (cover∼ns(year,4)	14	−65.0919	142.1838	0.0887	2.0170
	Regression (cover ∼year)	17	−66.4664	138.9328	0.4506	4.8072
	Polynomial (cover∼poly(year,4))	14	−64.5737	141.1474	0.1489	4.0433
Central	Intercept only (cover ∼1)	18	−71.2748	146.5495	0.3635	0.0000
	Spline (cover∼ns(year,4)	14	−68.7149	149.4298	0.0861	25.6366
	Regression (cover ∼ year)	17	−70.0061	146.0122	0.4756	3.3933
	Polynomial (cover∼poly(year,4))	14	−68.8562	149.7124	0.0748	3.5916
North	Intercept only (cover ∼1)	14	−58.8183	121.6366	0.0036	0.0000
	*Spline (cover∼ns(year,4)	**10**	−**50.4792**	**112.9585**	**0.2756**	**9.8881**
	*Regression (cover ∼ year)	**13**	−**53.0023**	**112.0046**	**0.4440**	**14.1852**
	*Polynomial (cover∼poly(year,4))	**10**	−**50.4748**	**112.9497**	**0.2768**	**14.1777**
North-east	Intercept only (cover ∼1)	10	−55.1855	114.3711	0.0049	0.0000
	*Spline (cover∼ns(year,4)	**6**	−**46.4170**	**104.8339**	**0.5803**	**8.5659**
	*Regression (cover ∼ year)	**9**	−**50.4584**	**106.9168**	**0.2048**	**14.0473**
	*Polynomial (cover∼poly(year,4))	**6**	−**47.4334**	**106.8669**	**0.2100**	**15.8892**
South	Intercept only (cover ∼1)	10	−51.7071	107.4142	0.0020	0.0000
	*Spline (cover∼ns(year,4)	**6**	−**42.1374**	**96.2748**	**0.5157**	**12.3751**
	*Regression (cover ∼ year)	**9**	−**45.3083**	**96.6166**	**0.4347**	**13.9038**
	Polynomial (cover∼poly(year,4))	6	−44.5179	101.0357	0.0477	18.5075

For each of the models contrasted, degrees of freedom (df), maximum log-likelihood (-*LL*), AIC*_c_*, AIC weight (*_w_*AIC*_c_*), and the % deviation explained (%DE) are shown. Splines are natural cubic, and Polynomials are 4^th^ order. Models in bold with * have significant relationships (<0.05).

**Table 3:** Results of the relationship between coral cover at Ningaloo Reef and explanatory variables using general linear mixed-effects model comparison based on Akaike’s information criterion (AIC*_c_*) corrected for small sample sizes.

Model	*df*	*–LL*	AIC*_c_*	*_w_*AIC*_c_*	%DE
Intercept only (cover ∼1)	4	−774.9794	1557.9587	0.0000	0.0000
Slope (year∼cover)	23	−747.6121	1541.2243	0.0000	3.5314
Habitat (year∼hab)	27	−736.8372	1527.6745	0.0000	4.9217
Area (year∼cover+area)	24	−753.02	1554.0400	0.0000	2.8335
Latitude (year∼cover+ lat)	57	−691.0784	1496.1568	0.9097	10.8262
All (year∼cover+area+hab+lat)	62	−688.3889	1500.7778	0.0903	11.1733

All models include the random effect ‘sampling site’. For each of the models contrasted, maximum log-likelihood (-*LL*), AIC*_c_*, AIC weight (*_w_*AIC*_c_*), and the % deviation explained (%DE) are shown.**.**

**Figure 4:**
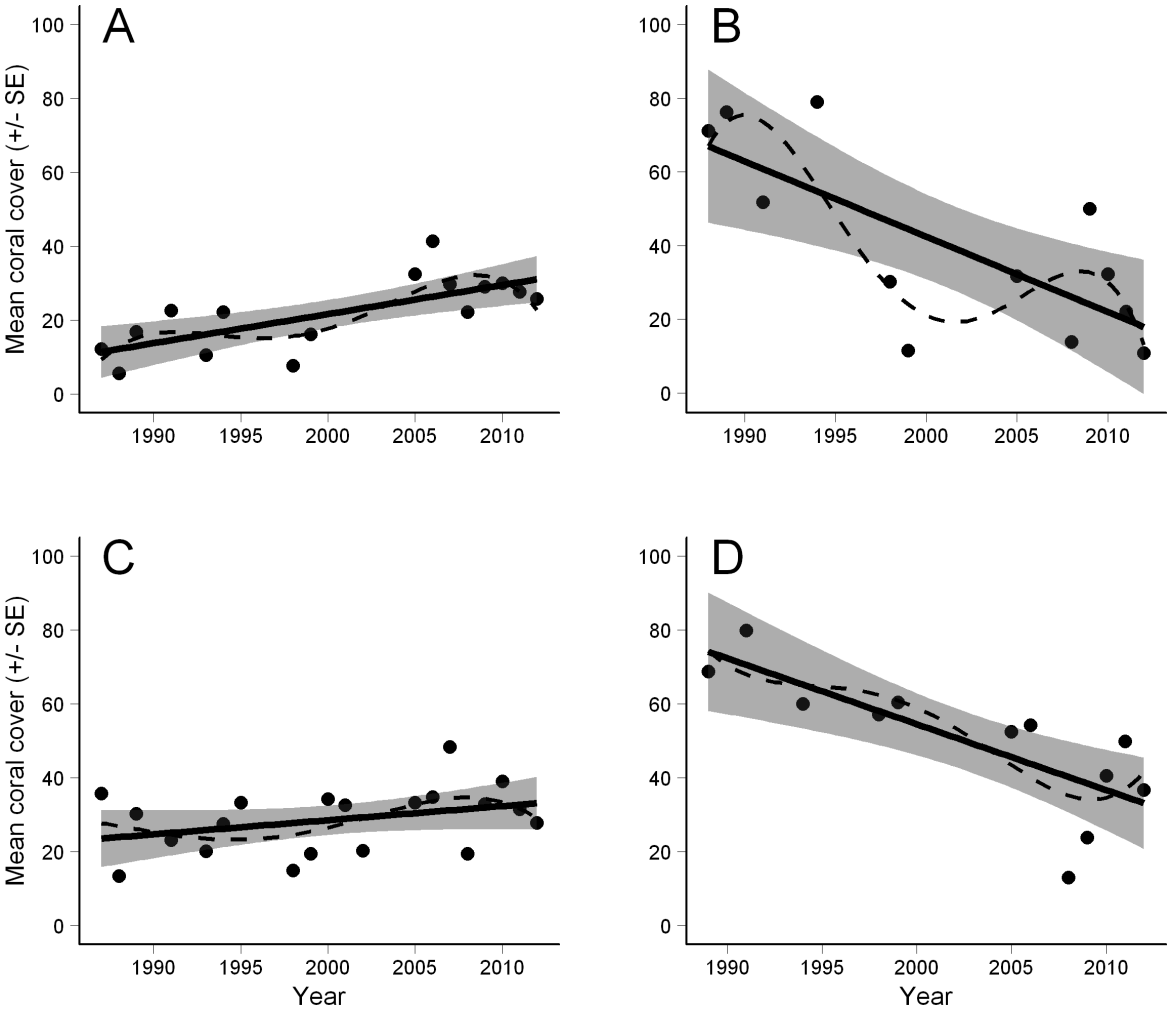
Coral cover through time at Ningaloo Reef weighted by area sampled (± SE). Lines fitted using a linear regression (solid line) and 4^th^ order polynomial regression (dashed line). A) North, B) North-east, C) Central, and D) South.

Sub-regions within Ningaloo Reef showed contrasting patterns in coral cover through time, with increasing coral cover in the North sub-region (Fig. 4a), and declines in the North-east and South sub-regions (Fig. 4b & d). In the Central sub-region the linear model had similar AIC*_c_* values to the intercept only model, suggesting no strong linear trends through time (Fig. 4c). However, in the North sub-region the linear model is the simplest and best explanatory model with a low AIC_c_ value and the most weight (*_w_*AIC*_c_* = 0.44). Linear and spline functions have similar AIC*_c_* and *_w_*AIC*_c_* values when modeling data from the south sub-region (Table 2), although there is clearly more weight for non-linear relationships (splines) in the North-east (*_w_*AIC*_c_* = 0.58) sub-region.

**Figure 5:**
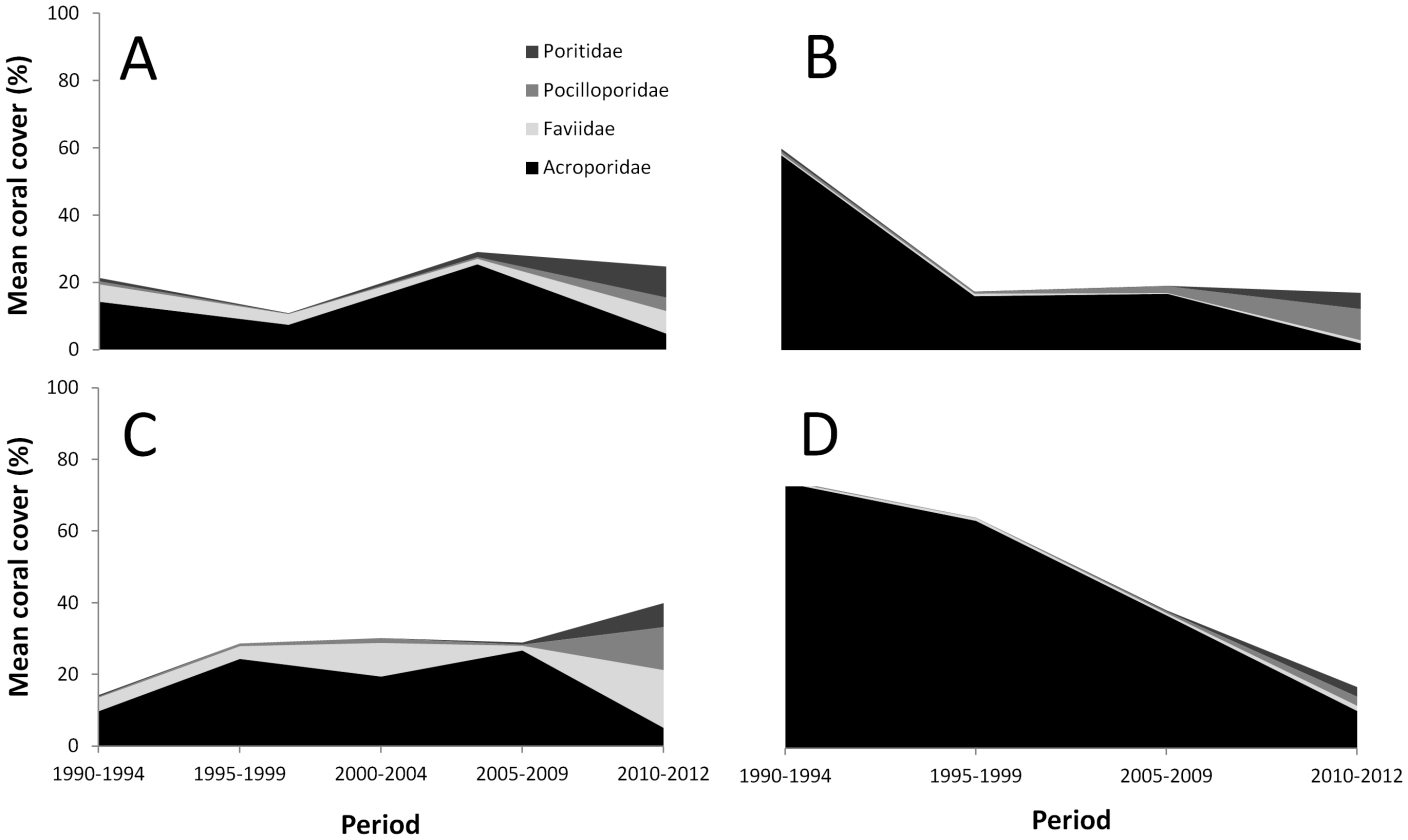
Coral community composition at Ningaloo Reef. **A) North, B) North-east, C) Central, and D) South.**

All Ningaloo Reef sub-regions were dominated by corals from the family Acroporidae, which declined in relative composition in the South sub-region (Adj. R^2^ = 0.94, P = 0.02) and the North-east sub-region, albeit a non-significant decrease (Adj. R^2^ = 0.70, P = 0.11) (Fig. 5b & d and Table S3). Since 2005, there has been some evidence to suggest that relative contributions of other coral families have increased, particularly in the Central sub-region (Fig. 5c).

## Discussion

Distribution and coral community composition on reefs are a function of environmental gradients [70], which are partly shaped by natural and anthropogenic pressures [20]. We used a meta-analytical approach to quantify these broad-scale patterns in coral cover for the south-eastern seaboard of the Indian Ocean, with the intent of assessing previous impacts and establishing benchmarks to be used for conservation and management. We identified that tropical coral reef habitats (i.e., north of Shark Bay) had average coral cover that ranged between 19.5% and 41%, which is comparable to other regions within the Indian Ocean [range 12%−42% 1] and [8] ∼25–35%, as well as averages on the Great Barrier Reef (GBR) [22% - 29% 3,4]. In comparison, temperate reef systems, marginal reefs, and areas of solitary corals (i.e., south of Shark Bay) ranged in average coral cover between ∼3% and 44%. This wide range in cover at higher latitudes reflects the patchy nature of coral communities in temperate environments. In the south -eastern Indian Ocean, corals in temperate waters are most prominent in areas more exposed to the warm waters of the Leeuwin current (e.g., reefs around the Abrolhos). Higher latitude regions generally also have coral communities of lower diversity than tropical regions due to fewer species being adapted to cooler climates [71], although an examination of coral diversity was beyond the scope of our study.

We found that while there was no clear relationship between latitude and coral cover at the family level for WA, there were notable differences in relation to community composition in coastal regions. The families Acroporidae and Poritidae showed greater relative contributions in the tropical regions compared to areas south of Shark Bay (within South-west inshore region), although Acroporidae was also prominent at the Abrolhos Islands [72], reflecting the influence of the Leeuwin current on these offshore islands. Similar latitudinal patterns in Acroporidae and Poritidae have been observed on the Great Barrier Reef [73], and it has been suggested that the decrease in presence of massive Poritidae colonies at high latitudes might be due to reduced growth [74], and accretion [73], which are related to unfavourable lower water temperatures [75]. Fewer numbers of Acroporidae species at higher latitude reefs has previously been observed [e.g., 73,76,77], and might be linked to this family being less tolerant to environmental stressors in marginal conditions when compared to other families such as Faviidae [70]. Reef accretion decreases dramatically south of the Abrolhos Islands [78], [79], which is similar to latitudes in South Africa (28° S), although these South African reefs are not true accretive reefs [80]. In comparison, reef development generally ceases around 24° S on the GBR [81].

Another notable distribution pattern along the WA coast was the prominence of members from the family Dendrophylliidae around the central WA inshore areas. In WA, this family was dominated by *Turbinaria* spp., and is distributed from the North offshore region to the Recherche Archipelago, which is located in the south-east of WA [37]. This genus is tolerant of inshore, turbid environments [82] and its success is likely due to their high sediment tolerance and ability to use detritus as a source of nutrients [82], [83]. This family of corals also appears well suited to higher latitude inshore areas on the GBR, which are characterised by cool, highly turbid waters [e.g., 73,81].

Despite fluctuations in total coral cover at Ningaloo Reef since the late 1980s, overall cover appears stable through time. This phenomenon has previously been described on the GBR as ‘dynamic stability’ [3]. A recent analysis of coral data from the GBR suggests coral cover is currently 14% and has declined, on average, by 0.5% per year between 1985 and 2012 [17]. Similarly, a meta-analysis of Indian Ocean reefs found that between 1968 and 2004 coral cover was decreasing at 1% per year [1] and in the Caribbean the decline is 1.5% per year [14] over a similar time period. The spatial scale of our study differs considerably to that of the aforementioned studies, so any comparisons of trends in coral cover should be made with caution. Indeed, comparing coral cover between, and within regions might often miss changes at the sub-regional level [2]. However, the maintenance of coral cover about a mean level of 28% over the past two and half decades when coral cover in other regions is declining is encouraging for Ningaloo Reef. Fluctuations in coral cover, with no general declining trend at Ningaloo is reflective of a system that has little to no chronic anthropogenic pressure in the form of agricultural runoff or heavy commercial fishing over the past 25 years, especially when compared to other large reef systems like the GBR [84], [85]. Indeed, a recent study at an isolated reef within the North offshore region found that recovery after natural disturbances (bleaching and cyclone disturbance) was rapid, and the authors suggested this was assisted by a lack of chronic anthropogenic pressures [86]. Despite the apparent stability in coral cover at Ningaloo Reef in recent times, we may actually be monitoring changes in an already significantly altered baseline, as has been observed through palaeoecological reconstructions on the GBR [87].

Peaks and troughs in coral cover at Ningaloo Reef were most prominent in the North-east region and can be related to acute environmental events. Exposure to prevailing wave energy in the North-east region is low and reefs in the region have no prominent reef front, so when cyclonic storms do come in close proximity to the reef the effects can be devastating [46]. A major trough in cover occurred around 1998/1999, which was evident across all Ningaloo sub-regions apart from the south. It is likely that multiple stressors affected coral cover during this period, with higher than average sea surface temperatures recorded across the Indian Ocean [30], and the occurrence of a destructive cyclone (“Vance”, category 4) crossing the Gulf of Exmouth in 1999. Although no bleaching data exist for Ningaloo during this period, wide spread bleaching was reported for the Indian Ocean [88]. Similarly, the combined effects of bleaching and cyclones have recently caused coral decline at Ningaloo, with up to 92% and 32% coral mortality on reefs in the North-east and South sub-regions, respectively [46], [47]. However, it seems unlikely that the decline in the South sub-region of Ningaloo Reef is as dramatic as our results suggest, as it is probable that areas of particularly high cover were targeted by earlier studies in the 1980s. In other sub-regions, localised declines in coral cover have been attributed to acute pressures such as coral spawn slicks that have caused anoxic events at Coral Bay [89], as well as outbreaks of the corallivorous gastropod *Drupella* spp. [90]. Short-term and localised declines in coral at Ningaloo Reef are, however, not attributable to outbreaks of crown-of-thorns starfish, which are one of the main drivers of coral loss in other parts of the world [e.g., 3,17,91].

The recent reduction of Acroporidae corals at Ningaloo may relate to the occurrence of pressures that typically affect this family more than other families, such as cyclones [92], predation by *Drupella* [93], disease [49] and bleaching [31]. Acroporidae corals are generally quick to recover after disturbances, provided sufficient cover remains, due to rapid growth rates and recolonisation [94], [95], although, if disturbances are frequent, other more robust families/morphologies might become relatively abundant. For example, in light of dramatic decreases in Acroporidae throughout the Carribean since the 1980s, other more resilient species such as *Porites astreoides* have become increasingly abundant [96], [97], [98]. Acroporidae corals are important habitat for juvenile fishes on reefs in WA [99], and declines in coral may influence the abundance of new recruits, with flow on effects for diversity and adult stocks [100], [101]. Furthermore, a decline in coral might result in reduced structural complexity on reefs which can reduce the diversity of fish communities [102]. There are also a high number of coral-feeding fish that feed specifically on corals from the family Acroporidae which, if reduced, could affect abundance and physiology of these fishes [103]. Many species of reef associated invertebrates also have specific preferences for Acroporidae [26], which can play important roles in herbivory, nutrient cycling, and water quality regulation [104].

Latitude explained more of the variance in models than habitat type or the size of area surveyed at Ningaloo Reef. This is possibly due to the majority of sampling sites being in the backreef, whereas considerable differences in total cover would normally be expected among reef crests, slopes and flats [e.g., 105]. Other studies have also found no significant correlation in coral cover with latitude [70], [73], which suggests that latitude by itself might not act as a good proxy for other variables such as water temperature, especially when currents transport warm waters to temperate latitudes. Overall, there was low support for models (∼10% Deviance Explained), which also suggests that other factors might be more important in determining coral cover such as: depth, water temperature, current regimes, or disturbances. Although we limited data to sites that were <20m, it is possible that pooling sites across this depth range might have obscured fine-scale difference in coral cover [e.g., 106].

Reviewing the available literature and datasets on coral cover and community composition along the WA coast has provided a means of identifying gaps in current knowledge. At present, taxonomically detailed and quantitative information is required for the Recherche Archipelago in the south, the Abrolhos Islands, and the Kimberley region in the north. Recent work in the Kimberley is starting to provide new information on species compositions for some reefs [107]. Understanding the current status of these communities and others is important for assessing shifts in distributions and understanding of resilience in light of predicted global changes in the marine environment [76], [108]. Southern regions in WA are likely to become increasingly important in detecting trends, as range extensions might occur with climate change. Indeed, recent evidence suggests that range extensions are already occurring for marine organisms, increasing the tropicalisation of temperate communities in Australia [76], [109], [110]. Furthermore, there appears to be sufficient substrate to support a latitudinal shift of corals further south in WA [51]. The overall lack of historical information for the extreme northern areas of WA is mostly attributable to its remoteness, lack of accessibility and low levels of anthropogenic pressure in this region making reefs there a lower monitoring priority.

Our study has highlighted the importance of assessing natural resources at multiple spatial scales along the WA coast. At the regional scale, Ningaloo Reef coral communities appear to be relatively stable through time, although at the sub-regional scale there are declining trends in the North-east and South that may require greater scrutiny and monitoring. On the east coast of tropical Australia, coral cover on some sections of the GBR have remained stable, although there has been an overall decline in coral cover over the past several decades [17]. Ningaloo lacks some of the key factors that are thought to be responsible for this decline on the more heavily populated coast of the GBR, namely, large-scale terrestrial runoff and outbreaks of crown-of-thorns starfish. As such, Ningaloo and other coral reefs of northwest WA may play an instructive role in assessing the relative importance of local versus global effects on coral cover. Further research is required to provide a complete understanding of coral distribution and community composition along the west Australian coastline. Such information would provide a robust baseline for assessing broader-scale impacts of climate change or trends in coral cover. Congruence in routine monitoring methods, combined with more collaboration between management agencies, industry, academic and scientific institutions would also facilitate broad-scale assessments required for comprehensive understanding, allowing more effective management and conservation decisions to be made.

## Supporting Information

Figure S1 **Correlograms of time-series coral cover data for Ningaloo Reef: A) before inclusion of correlation structure in mixed-effects model, and B) after inclusion of correlation structure.**
(DOCX)

Table S1 **List of documents and sources where data were derived for meta-analysis.** Note: Analysis of photo and video transects were done in the lab, as opposed to visual assessments, quadrats and *in-situ* point or line intercept methods, which were done in the field at the time of sampling.
(DOCX)

Table S2 **Results of linear regression to assess relationship between latitude and coral cover by dominant families of corals off the Western Australian coast.**
(DOCX)

Table S3 **Results of linear regression to assess relationship between coral cover of dominant families through time at each sub-region of Ningaloo Reef.**
(DOCX)

Table S4 **Checklist of items done during the meta-analysis process.**
(DOCX)
