# Supplementary material for: Dynamic Stability of Coral Reefs on the West Australian Coast
Source: PLoS One. 2013 Jul 29;8(7):e69863. doi: 10.1371/journal.pone.0069863 (PMC3726730; doi:10.1371/journal.pone.0069863)

Figure S1. Correlograms of time-series coral cover data for Ningaloo Reef: A) before inclusion of correlation structure in mixed-effects model, and B) after inclusion of correlation structure.


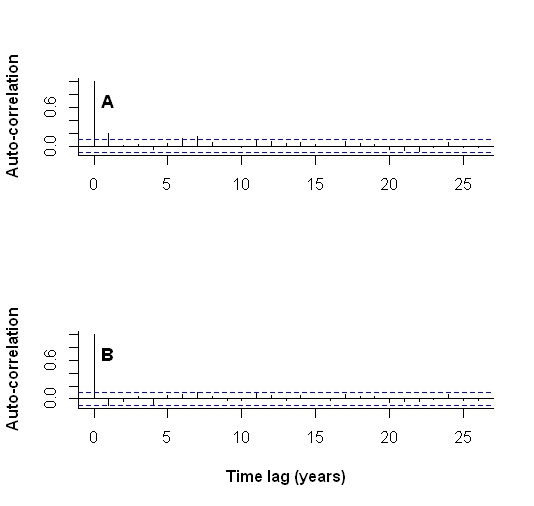

Supplement: Figure S1 — Correlograms of time-series coral cover data for Ningaloo Reef: A) before inclusion of correlation structure in mixed-effects model, and B) after inclusion of correlation structure. (DOCX) [file pone.0069863.s001.docx]
