# Supplementary material for: Dynamic Stability of Coral Reefs on the West Australian Coast
Source: PLoS One. 2013 Jul 29;8(7):e69863. doi: 10.1371/journal.pone.0069863 (PMC3726730; doi:10.1371/journal.pone.0069863)
Supplement: Table S1 — List of documents and sources where data were derived for meta-analysis. Note: Analysis of photo and video transects were done in the lab, as opposed to visual assessments, quadrats and in-situ point or line intercept methods, which were done in the field at the time of sampling. (DOCX) [file pone.0069863.s002.docx]

Table S1. List of documents and sources where data were derived from for meta-analysis. Note: Analysis of photo and video transects were done in the lab, as opposed to visual assessments, quadrats and *in-situ* point or line intercept methods, which were done in the field at the time of sampling.

| **Study #** | **Year(s) collected** | **WA region** | **Collection method** | **Name of collector(s)** | **Data contact** | **Publication name** |
| --- | --- | --- | --- | --- | --- | --- |
| 1 | 2005 & 2009 | North Offshore | *In-situ* point-intercept transects | Ceccarelli et al. | School of Marine & Tropical Biology, James Cook University Townsville QLD 4811 Australia | Ceccarelli D, Richards Z, Pratchett M, Cvitanovic C (2011) Rapid increase in coral cover on an isolated coral reef, the Ashmore Reef National Nature Reserve, north-western Australia. Marine and Freshwater Research 62:1214-1220 |
| 2 | 1994-1998, 2001 & 2003 | North Offshore | Visual assessments | Rees et al. | Australian Institute of Marine Science, The UWA Oceans Institute (M096), 35 Stirling Hwy Crawley6009, Western Australia, Australia | Rees, M., H. Australia. Dept. of the Environment and, et al. (2003). Surveys of Trochus, Holothuria, Giant Clams, and the Coral Communities at Ashmore Reef, Cartier Reef, and Mermaid Reef, Northwestern Australia, Australian Institute of Marine Science. |
| 3 | 1996 - 1998, 2002 & 2005 | North Offshore | Video point-intercept transects | AIMS | Australian Institute of Marine Science, The UWA Oceans Institute (M096), 35 Stirling Hwy Crawley6009, Western Australia, Australia | AIMS (2006). Coral reef communities at the Rowley Shoals, north-western Australia: state of knowledge and management implications. Perth, Australian Institute of Marine Science. |
| 4 | 2004 | Pilbara inshore | Video point-intercept transects | Stoddard et al. | Mscience, 99 Broadway, Nedlands WA 6009, Australia | Stoddart, J. A., K. A. Grey, et al. (2004). "Rapid high-precision monitoring of coral communities to support reactive management of dredging in Mermaid Sound, Dampier, Western Australia." Corals of the Dampier Harbour: Their Survival and Reproduction During the Dredging Programs of: 35-52. |
| 5 | 1998 | North Offshore | Visual assessments | Skewes et al. | Commonwealth Scientific and Industrial Research Organisation, Centre for Environment and Life Sciences - Floreat WA, Underwood Avenue, Floreat WA 6014, Australia | Skewes, T. D., D. M. Dennis, et al. (1999). Survey and Stock Size Estimates of the Shallow Reef (0-15 m Deep) and Shoal Area (15-50 m Deep) Marine Resources and Habitat Mapping Within the Timor Sea MOU74 Box - Volume 2: Habitat mapping and coral dieback, CSIRO Marine Research. |
| 6 | 2001 | North Offshore | Video point-intercept transects | Gilmour et al. | Australian Institute of Marine Science, The UWA Oceans Institute (M096), 35 Stirling Hwy Crawley6009, Western Australia, Australia | Gilmour et al. (2007) Data compilation and analysis for Rowley Shoals Mermaid, Imperieuse and Clerke reefs. Prepared for DEWR. |
| 7 | 1995-2000, 2002, & 2004 | North Offshore | Video point-intercept transects | Smith et al. | Australian Institute of Marine Science, The UWA Oceans Institute (M096), 35 Stirling Hwy Crawley6009, Western Australia, Australia | Smith, L. D., J. P. Gilmour, et al. (2008). "Resilience of coral communities on an isolated system of reefs following catastrophic mass-bleaching." Coral Reefs 27(1): 197-205. |
| 8 | 2001 & 2008 | North Offshore | Video point-intercept transects | AIMS | Australian Institute of Marine Science, The UWA Oceans Institute (M096), 35 Stirling Hwy Crawley6009, Western Australia, Australia | Woodside (2008) Scott Reef Status Report 2008 |
| 9 | 1993 | North Offshore | Video point-intercept transects | Done et al. | Australian Institute of Marine Science, The UWA Oceans Institute (M096), 35 Stirling Hwy Crawley6009, Western Australia, Australia | Done, T. J., D. M. B. Williams, et al. (1994). "Surveys of coral and fish communities at Scott Reef and Rowley Shoals." Australian Institute of Marine Sciences 46. |
| 10 | 1987 | Ningaloo Reef | Visual assessments | Ayling & Ayling | Marine Science Program, West Australian Department of Environment and Conservation, 17 Dick Perry Avenue KensingtonLocked Bag 104,Bentley Delivery Centre 6983 | Ayling, T., A. L. Ayling, et al. (1987). Ningaloo Marine Park: Preliminary Fish Density Assessment and Habitat Survey: with Information on Coral Damage Due to *Drupella Cornus* Grazing: a Report Prepared for the Department of Conservation and Land Management, Western Australia, Department of Conservation and Land Management. |
| 11 | 2009 | Ningaloo Reef | *In-situ* point-intercept transects | Johansson et al. | School of Marine & Tropical Biology, James Cook University Townsville QLD 4811 Australia | Johansson, C. L., D. R. Bellwood, et al. (2010). "Sea urchins, macroalgae and coral reef decline: a functional evaluation of an intact reef system, Ningaloo, Western Australia." Marine Ecology Progress Series 414: 65-74. |
| 12 | 2009 | Ningaloo Reef | *In-situ* point-intercept transects | Johansson et al. | School of Marine & Tropical Biology, James Cook University Townsville QLD 4811 Australia | Johansson CL, Bellwood DR, Depczynski M (In Press) The importance of live coral for small sized herbivorous reef fishes in physically challenging environments |
| 13 | 2008 | Ningaloo Reef | *In-situ* line-intercept transects | Verges et al. | Sydney Institute of Marine Science and Evolution and Ecology Research Centre, School of Biological, Earth and Environmental Sciences, University of New South Wales, Sydney, Australia | Verges, A., M. A. Vanderklift, et al. (2011). "Spatial patterns in herbivory on a coral reef are influenced by structural complexity but not by algal traits." PLoS One 6(2): e17115. |
| 14 | 2002 | Ningaloo Reef | Visual assessments | Fitzpatrick & Penrose | Oceanwise, Perth, Western Australia | Fitzpatrick, B. and H. Penrose (2002). A preliminary marine ecological survey of Bateman Bay, Ningaloo Reef, Oceanwise & Murdoch University. |
| 15 | 1989 | Ningaloo Reef | *In-situ* line-intercept transects | Simpson et al. | Marine Science Program, West Australian Department of Environment and Conservation, 17 Dick Perry Avenue KensingtonLocked Bag 104,Bentley Delivery Centre 6983 | Simpson, C. J., J. L. Cary, et al. (1993). "Destruction of corals and other reef animals by coral spawn slicks on Ningaloo Reef, Western Australia." Coral Reefs 12(3): 185-191. |
| 16 | 1985 | Pilbara inshore | *In-situ* line-intercept transects | Simpson & Grey | Marine Science Program, West Australian Department of Environment and Conservation, 17 Dick Perry Avenue Kensington Locked Bag 104,Bentley Delivery Centre 6983 | Simpson, C. J. and K. A. Grey (1989). Survey of Crown-of-Thorns starfish and coral communities in the Dampier Archipelago, Western Australia. Western Australian Environmental Protection Authority, Technical Series 25. Technical Series 25: 1–24. |
| 17 | 2005 | Ningaloo Reef | Video point-intercept transects | Armstrong, S. J. | Southern Cross, PO Box 157 Lismore NSW 2480 | Armstrong, S.J. (2005). The abundance and distribution of *Drupella* corallivourus gastropods at Ningaloo Reef, Western Australia. Honours Thesis, Southern Cross, University. |
| 18 | 1994 | Ningaloo Reef | Quadrats | Osborne & Williams | Marine Science Program, West Australian Department of Environment and Conservation, 17 Dick Perry Avenue KensingtonLocked Bag 104,Bentley Delivery Centre 6983 | Osborne, S. & Williams, M.R. (1995). Status of *Drupella cornus* outbreak at Ningaloo Reef. Final report prepared for the Australian Nature Conservation Agency. Department of Conservation and Land Management, Perth, Western Australia. |
| 19 | 1996 | Shark Bay | Video point-intercept transects | Cary, J. L. | Marine Science Program, West Australian Department of Environment and Conservation, 17 Dick Perry Avenue Kensington Locked Bag 104,Bentley Delivery Centre 6983 | Cary J L (1997), Baseline studies and monitoring of visitor sites in the Shark Bay Marine Park, Shark Bay World Heritage Area and Hamelin Pool Marine Nature Reserve, Shark Bay marine reserves monitoring programme: final report MMSP/MW/SBMP-4/1997, Commonwealth Department of Tourism and Department of Conservation and Land Management, Perth, Western Australia, 35. |
| 20 | 2006 | Pilbara offshore | Video point-intercept transects | Bancroft, K. P. | Marine Science Program, West Australian Department of Environment and Conservation, 17 Dick Perry Avenue Kensington Locked Bag 104,Bentley Delivery Centre 6983 | Bancroft KP (2011) Long-term coral community monitoring in the Montebello/Barrow Islands marine protected areas: site descriptions and summary analysis of baseline data collected in December 2006 Marine Science Program Data Report, MSPDR9, June 2011. Department of Environment and Conservation, Perth, Western Australia |
| 21 | 2008 | Pilbara inshore | Video point-intercept transects | SKM | Sinclair Knight Merz, 11th Floor, Durack Centre, 263 Adelaide Terrace, Perth, WA 6000 | SKM (2009) Port Hedland outer harbour development: Baseline coral health monitoring report periods 1-13. |
| 22 | 2009 | Kimberley | Photo point-intercept transects | Heyward & Moore | Australian Institute of Marine Science, The UWA Oceans Institute (M096), 35 Stirling Hwy Crawley6009, Western Australia, Australia | Heyward, A. and C. Moore (2009). Benthic habitat characterisation of Montgomery Reef: Assessing the distribution and relative abundance of dominant benthic communities FIELD REPORT - 2009, Australian Institute of Marine Science. |
| 23 | 2011 | South-west inshore | Photo point-intercept transects | Hunter, H. | Murdoch University, 90 South Street, Murdoch WA 6150, Australia | Hunter, H. (2011). Hard Corals of Hall Bank, Western Australia - Honours Thesis, Murdoch University. |
| 24 | 2009 | Pilbara inshore | *In-situ* line-intercept transects | WorleyParsons | WorleyParsons, L 7, 250 St Georges Tce, PERTH, WA, 6000 | WorleyParsons (2009). Comparison of the Dampier Port Fringing Reef Benthic Community with Nearby Reef Areas. Perth: 1-29. |
| 25 | 2008 | Pilbara inshore | Photo point-intercept transects | MScience | Mscience, 99 Broadway, Nedlands WA 6009, Australia | MScience (2008). Cape Lambert Benthic Habitat Mapping Report: MSA123R1 MScience Pty Ltd, 239 Beaufort St, Perth, WA 6003, Australian Benthic Marine habitats at Cape Lambert West, Bezout, Dixon and Delambre Islands. Perth, Report to Maunsell Australia Pty Lty. |
| 26 | 2009 | Pilbara inshore | Photo point-intercept transects | MScience | Mscience, 99 Broadway, Nedlands WA 6009, Australia | MScience (2009). Wheatstone LNG Development: Baseline coral community description, Report to URS Australia. |
| 27 | 2009 | South-west inshore | Video point-intercept transects | SKM | Fremantle Ports, PO Box 95Fremantle WA 6959 | SKM (2011). Fremantle Ports Inner Harbour Deepening and Reclamation – Post Dredging Coral Monitoring Report : Coral monitoring program Hall Bank, Sinclair Knight Merz. |
| 28 | 2008 | Pilbara inshore | *In-situ* point-intercept transects | SKM | Sinclair Knight Merz, 11th Floor, Durack Centre, 263 Adelaide Terrace, Perth, WA 6000 | SKM (2008). Cape Lambert Port B Development - Abundance and distribution of inter and subtidal benthic habitats in the Cape Lambert area: 2008 survey. Perth, Sinclair Knight Merz. |
| 29 | 2008 | Offshore Territories | Visual assessments | Hobbs, J-P. A. | School of Marine & Tropical Biology, James Cook University Townsville QLD 4811 Australia | Hobbs, Jean-Paul A. (2011) Reef fishes on isolated islands: community structure, endemism and extinction. PhD thesis. James Cook University. |
| 30 | 1998 | Pilbara inshore | Video point-intercept transects | Morrison, P. F. | WA Museum, Locked Bag 49, Welshpool DC. WA 6986 | Morrison PF (2004) A general description of the subtidal habitats of the Dampier Archipelago, Western Australia. Records of the Western Australian Museum Supplement No. 66: 51-59. |
| 31 | 2007 | Pilbara inshore | Video point-intercept transects | Mscience | Mscience, 99 Broadway, Nedlands WA 6009, Australia | Mscience (2007) Pluto LNG Development-Coral health monitoring: baseline. |
| 32 | 2010 | Abrolhos | Video point-intercept transects | Smale et al. | University of Western Australia, 35 Stirling Hwy Crawley WA 6009 | Smale DA, Kendrick GA, Harvey ES, Langlois TJ, Hovey RK, Van Niel KP, Waddington KI, Bellchambers LM, Pember MB, Babcock RC (2012) Regional-scale benthic monitoring for ecosystem-based fisheries management (EBFM) using an autonomous underwater vehicle (AUV). ICES Journal of Marine Science: Journal du Conseil 69:1108-1118 |
| 33 | 2006 | North Offshore | Video point-intercept transects | Sampey & Fromont | WA Museum, Locked Bag 49, Welshpool DC. WA 6986 | Sampey & Fromont (2011) Patterns in marine community assemblages on continental margins: a faunal and floral synthesis from northern Western Australian atolls. Journal of the Royal Society of Western Australia, 94(2). |
| 34 | 1999 | South-west inshore | *In-situ* point-intercept transects | Edgar et al. | Institute for marine and Antarctic studies, Sandy Bay Cnr Alexander Street & Grosvenor Street Sandy Bay Tasmania Australia | Edgar G, Barrett N, Crane K, Bancroft K. (2007) Ecosystem monitoring of subtidal reefs in different management zones of the Jurien Bay Marine Park 1999–2007. TAFI internal report. |
| 35 | 2004 | Abrolhos | Video point-intercept transects | Dinsdale & Smith | West Australian Department of Fisheries, WA Fisheries and Marine Research Laboratories, 39 Northside Drive, Hillarys WA 6025, Australia | Dinsdale E & Smith L (2004) Broadscale survey of coral condition on the reefs of the Easter Group of the Houtman Abrolhos Islands. West Aust Dept Fisheries Internal, AN: 17292 (1). |
| 36 | 1980 | Ningaloo Reef | Visual assessments | WA Museum | WA Museum, Locked Bag 49, Welshpool DC. WA 6986 | Unpublished |
| 37 | 1995 | Ningaloo Reef | Video point-intercept transects | AIMS | Australian Institute of Marine Science, The UWA Oceans Institute (M096), 35 Stirling Hwy Crawley6009, Western Australia, Australia | Unpublished |
| 38 | 1999 | Ningaloo Reef | Video line-intercept transects | Grubba & Cary | Marine Science Program, West Australian Department of Environment and Conservation, 17 Dick Perry Avenue KensingtonLocked Bag 104,Bentley Delivery Centre 6983 | Grubba TL, Cary JL (2000) Survey of the monitoring sites established in 1989 after coral mortality in Bills Bay from the coral mass spawning event of March 1989. Marine Conservation Branch, Department of Conservation and Land Management, Perth, Western Australia |
| 39 | 2002 | Ningaloo Reef | Video point-intercept transects | AIMS | Australian Institute of Marine Science, The UWA Oceans Institute (M096), 35 Stirling Hwy Crawley6009, Western Australia, Australia | Unpublished |
| 40 | 2006 | Ningaloo Reef | Video point-intercept transects | DEC | Marine Science Program, West Australian Department of Environment and Conservation, 17 Dick Perry Avenue Kensington Locked Bag 104,Bentley Delivery Centre 6983 | Unpublished |
| 41 | 2011 | Ningaloo Reef | Video point-intercept transects | DEC | Marine Science Program, West Australian Department of Environment and Conservation, 17 Dick Perry Avenue Kensington Locked Bag 104,Bentley Delivery Centre 6983 | Unpublished |
| 42 | 1988 | Ningaloo Reef | *In-situ* line-intercept transects | Forde, J. M. | Marine Science Program, West Australian Department of Environment and Conservation, 17 Dick Perry Avenue KensingtonLocked Bag 104,Bentley Delivery Centre 6983 | Forde JM (1994) Ecology of the muricid gastropod *Drupella cornus* (Roding, 1798) and its significance as a corallivore on Ningaloo reef, Western Australia. Master of Science thesis, University of Western Australia, p 100 |
| 43 | 1989 | Ningaloo Reef | Video line-intercept transects | Osborne & Williams | Marine Science Program, West Australian Department of Environment and Conservation, 17 Dick Perry Avenue Kensington Locked Bag 104,Bentley Delivery Centre 6983 | Unpublished |
| 44 | 1991 | Ningaloo Reef | Video line-intercept transects | Osborne & Williams | West Australian Department of Environment and Conservation, 17 Dick Perry Avenue Kensington Locked Bag 104,Bentley Delivery Centre 6983 | Unpublished |
| 45 | 1993 | Ningaloo Reef | Video point-intercept transects | AIMS | Australian Institute of Marine Science, The UWA Oceans Institute (M096), 35 Stirling Hwy Crawley6009, Western Australia, Australia | Unpublished |
| 46 | 1998 | Ningaloo Reef | Video point-intercept transects | AIMS | Australian Institute of Marine Science, The UWA Oceans Institute (M096), 35 Stirling HwyCrawley6009, Western Australia, Australia | Unpublished |
| 47 | 1998 | Ningaloo Reef | Video line-intercept transects | DEC | Marine Science Program, West Australian Department of Environment and Conservation, 17 Dick Perry Avenue Kensington Locked Bag 104,Bentley Delivery Centre 6983 | Cary JL, Grubba TL, Myers J (1999) Ningaloo Marine Park Monitoring program: benthic monitoring sites established in 1998 - data report. CALM, Perth |
| 48 | 1999 | Ningaloo Reef | Video point-intercept transects | AIMS | Australian Institute of Marine Science, The UWA Oceans Institute (M096), 35 Stirling Hwy Crawley6009, Western Australia, Australia | Unpublished |
| 49 | 2006 | Ningaloo Reef | Video point-intercept transects | DEC | Marine Science Program, West Australian Department of Environment and Conservation, 17 Dick Perry Avenue Kensington Locked Bag 104,Bentley Delivery Centre 6983 | DEC (2006) *Drupella* monitoring report. |
| 50 | 2009 | Ningaloo Reef | Video point-intercept transects | AIMS | Australian Institute of Marine Science, The UWA Oceans Institute (M096), 35 Stirling HwyCrawley6009, Western Australia, Australia | Unpublished |
| 51 | 2010 | Ningaloo Reef | Video point-intercept transects | DEC | Marine Science Program, West Australian Department of Environment and Conservation, 17 Dick Perry Avenue Kensington Locked Bag 104,Bentley Delivery Centre 6983 | Unpublished |
| 52 | 2011 | Ningaloo Reef | Video point-intercept transects | DEC | Marine Science Program, West Australian Department of Environment and Conservation, 17 Dick Perry Avenue Kensington Locked Bag 104,Bentley Delivery Centre 6983 | Unpublished |
| 53 | 2008 | Ningaloo Reef | Video point-intercept transects | DEC | Marine Science Program, West Australian Department of Environment and Conservation, 17 Dick Perry Avenue Kensington Locked Bag 104,Bentley Delivery Centre 6983 | DEC (2008) *Drupella* monitoring report. |
| 54 | 2009 | South-west inshore | Photo point-intercept transects | Thomson & Frisch | Commonwealth Scientific and Industrial Research Organisation, Centre for Environment and Life Sciences - Floreat WA, Underwood Avenue, Floreat WA 6014, Australia | Thomson, D. P. and A. J. Frisch (2010). "Extraordinarily high coral cover on a nearshore, high-latitude reef in south-west Australia." Coral Reefs 29(4): 923-927. |
| 55 | 2011 | South-west inshore | Photo point-intercept transects | Thomson, D. | Commonwealth Scientific and Industrial Research Organisation, Centre for Environment and Life Sciences - Floreat WA, Underwood Avenue, Floreat WA 6014, Australia | Unpublished |
| 56 | 2005 | South-west inshore | Quadrats | Thomson, D. | Commonwealth Scientific and Industrial Research Organisation, Centre for Environment and Life Sciences - Floreat WA, Underwood Avenue, Floreat WA 6014, Australia | Unpublished |
| 57 | 2010 | Abrolhos | Quadrats | WA Fisheries | West Australian Department of Fisheries, WA Fisheries and Marine Research Laboratories, 39 Northside Drive, Hillarys WA 6025, Australia | Unpublished |
| 58 | 2008 | North Offshore | Video point-intercept transects | AIMS | Australian Institute of Marine Science, The UWA Oceans Institute (M096), 35 Stirling Hwy Crawley6009, Western Australia, Australia | Woodside (2010). Scott Reef Status Report 2010, Woodside. |
| 59 | 2010 | Shark Bay | Video point-intercept transects | Bancroft, K. P. | Marine Science Program, West Australian Department of Environment and Conservation, 17 Dick Perry Avenue KensingtonLocked Bag 104,Bentley Delivery Centre 6983 | Unpublished |
| 60 | 2007 | Pilbara inshore | Video point-intercept transects | Armstrong, S. J. | Marine Science Program, West Australian Department of Environment and Conservation, 17 Dick Perry Avenue Kensington Locked Bag 104,Bentley Delivery Centre 6983 | Armstrong, S. J. (2009). Assessing the effectiveness of sanctuary zones in the proposed Dampier Archipelago Marine Park. Perth, Department of Environment and Conservation. |
| 61 | 2011 | Ningaloo Reef | *In-situ* line-intercept transects | Wilson et al. | Marine Science Program, West Australian Department of Environment and Conservation, 17 Dick Perry Avenue Kensington Locked Bag 104,Bentley Delivery Centre 6983 | Wilson, S. K., Babcock, R. C., Fisher, R., Holmes, T. H., Moore, J. A. Y., Thomson, D. P. (2012). Relative and combined effects of habitat and fishing on reef fish communities across a limited fishing gradient at Ningaloo. Marine Environmental Research81 : 1-11 |
| 62 | 2009 | Ningaloo Reef | Visual assessments | Depczynski et al. | West Australian Marine Science Institute, WAMSI Headquarters, Botany and Biology Building (M095), The University of Western Australia, 35 Stirling Highway, Crawley WA 6009 | Unpublished |
| 63 | 2007 | Ningaloo Reef | Photo point-intercept transects | Babcock et al. | Commonwealth Scientific and Industrial Research Organisation, Centre for Environment and Life Sciences - Floreat WA, Underwood Avenue, Floreat WA 6014, Australia | Babcock R, Vanderklift MA, Murphy N, Clapin G, limbourn A, Phillips J, Haywood M, Kozak D, Thomson DP, Cook K, Harriet D, Downie R, Parker F, Bearham D (2009) Ecosystem impacts of human usage and the effectiveness of zoning for biodiversity conservation trophic effects surveys final report. WAMSI, Perth |
| 64 | 2009 | Pilbara offshore | Video point-intercept transects | DEC | Marine Science Program, West Australian Department of Environment and Conservation, 17 Dick Perry Avenue Kensington Locked Bag 104,Bentley Delivery Centre 6983 | Unpublished |
| 65 | 2008 | Ningaloo Reef | Visual assessments | Depczynski et al. | Australian Institute of Marine Science, The UWA Oceans Institute (M096), 35 Stirling Hwy Crawley6009, Western Australia, Australia | Unpublished |
| 66 | 2007 | Kimberley | Visual assessments | INPEX | INPEX, Level 22100 St Georges TcePerth, WA 6000 | INPEX (2011) Biological and Ecological Studies of the Bonaparte Archipelago, Chapter 8: Marine Ecology (INPEX Doc Nr CO75-AH-REP-0027, Draft). Based on RPS (2008): INPEX Environmental Impact Studies Technical Appendix: Marine Ecology (Report No: M06604, Rev 1 of 1 October 2008). INPEX, Perth |
| 67 | 2010 | Kimberley | Visual assessments | Wilson et al. | WA Museum, Locked Bag 49, Welshpool DC. WA 6986 | Wilson B, Blake S, Ryan D, Hacker J (2001) Reconnaissance of species-rich coral reefs in a muddy, macro-tidal, enclosed embayment, - Talbot Bay, Kimberley, Western Australia. Journal of the Royal Society of Western Australia 94:251-265 |
| 68 | 2001 | Ningaloo Reef | Video point-intercept transects | AIMS | Australian Institute of Marine Science, The UWA Oceans Institute (M096), 35 Stirling Hwy Crawley6009, Western Australia, Australia | Unpublished |
| 69 | 2000 | Ningaloo Reef | Video point-intercept transects | DEC | Marine Science Program, West Australian Department of Environment and Conservation, 17 Dick Perry Avenue Kensington Locked Bag 104,Bentley Delivery Centre 6983 | Unpublished |
| 70 | 1999 | Ningaloo Reef | Video line-intercept transects | Cary et al. | Marine Science Program, West Australian Department of Environment and Conservation, 17 Dick Perry Avenue KensingtonLocked Bag 104,Bentley Delivery Centre 6983 | Cary JL, Grubba TL, Mahendran M, Radford B (2000) Ningaloo Marine Park Monitoring program: benthic monitoring sites established in 1999 - data report. CALM, Perth |
| 71 | 2009 | Ningaloo Reef | Video point-intercept transects | NA | Marine Science Program, West Australian Department of Environment and Conservation, 17 Dick Perry Avenue Kensington Locked Bag 104,Bentley Delivery Centre 6983 | Unpublished |
| 72 | 2007 | Ningaloo Reef | Quadrats | Kobryn et al. | Murdoch University, 90 South Street, Murdoch WA 6150, Australia | Unpublished ground truthing for Kobryn HT, Wouters K, Beckley, LE (2011)  Habitats and biodiversity of the Ningaloo Reef lagoon and adjacent coastal areas with hyperspectral imagery  Ningaloo Collaboration Cluster Final Report No.1b |
| 73 | 2005 | North Offshore | *In-situ* point-intercept transects | Australian Government of Department of Sustainability, Environment, Water Population and Communities | Australian Government of Department of Sustainability, Environment, Water Population and Communities, GPO Box 787 Canberra ACT 2601 Australia | Richards, Z., Beger, M., Hobbs, J. P., Bowling, T., Chong-Seng, K. and Pratchett, M. (2009). Ashmore Reef National Nature Reserve and Cartier Island Marine Reserve - Marine Survey 2009, Final Report, Department of the Environment, Water Heritage & the Arts. |
| 74 | 2009 | North Offshore | *In-situ* point-intercept transects | Australian Government of Department of Sustainability, Environment, Water Population and Communities | Australian Government of Department of Sustainability, Environment, Water Population and Communities, GPO Box 787Canberra ACT 2601 Australia | Richards, Z., Beger, M., Hobbs, J. P., Bowling, T., Chong-Seng, K. and Pratchett, M. (2009). Townsville, Produced for the Department of Environment, Water, Heritage, and the Arts. |
| 75 | 2010 | North Offshore | Video point-intercept transects | AIMS | Australian Institute of Marine Science, The UWA Oceans Institute (M096), 35 Stirling Hwy Crawley6009, Western Australia, Australia | Gilmour, J. P., L. D. Smith, et al. (2013). "Recovery of an Isolated Coral Reef System Following Severe Disturbance." Science 340 (6128): 69-71. |
| 76 | 2010 | South-west inshore | Photo point-intercept transects | Smale et al. | University of Western Australia, 35 Stirling Hwy Crawley WA 6009 | Smale DA, Kendrick GA, Harvey ES, Langlois TJ, Hovey RK, Van Niel KP, Waddington KI, Bellchambers LM, Pember MB, Babcock RC (2012) Regional-scale benthic monitoring for ecosystem-based fisheries management (EBFM) using an autonomous underwater vehicle (AUV). ICES Journal of Marine Science: Journal du Conseil 69:1108-1118 |
