# Supplementary material for: Dynamic Stability of Coral Reefs on the West Australian Coast
Source: PLoS One. 2013 Jul 29;8(7):e69863. doi: 10.1371/journal.pone.0069863 (PMC3726730; doi:10.1371/journal.pone.0069863)
Supplement: Table S2 — Results of linear regression to assess relationship between latitude and coral cover by dominant families of corals off the Western Australian coast. (DOCX) [file pone.0069863.s003.docx]

Table S2. Results of linear regression to assess relationship between latitude and coral cover by dominant families of corals off the Western Australian coast.

| **Family** | ***df*** | **Slope** | **F stat.** | **Adj. R^2^** | **P value** |
| --- | --- | --- | --- | --- | --- |
| Acroporidae | 5 | -0.6750 | 5.4250 | 0.4244 | 0.0673 |
| Dendrophylliidae | 5 | 0.1207 | 0.0794 | -0.1813 | 0.7895 |
| Faviidae | 5 | 0.0502 | 0.0188 | -0.1955 | 0.8962 |
| Pocilloporidae | 5 | -0.1506 | 1.9250 | 0.1336 | 0.2240 |
| Poritidae | 5 | -0.4954 | 6.0710 | 0.4581 | 0.0570 |
