# Supplementary material for: Dynamic Stability of Coral Reefs on the West Australian Coast
Source: PLoS One. 2013 Jul 29;8(7):e69863. doi: 10.1371/journal.pone.0069863 (PMC3726730; doi:10.1371/journal.pone.0069863)
Supplement: Table S3 — Results of linear regression to assess relationship between coral cover of dominant families through time at each sub-region of Ningaloo Reef. (DOCX) [file pone.0069863.s004.docx]

Table S3. Results of linear regression to assess relationship between coral cover of dominant families through time at each sub-region of Ningaloo Reef.

| **Region** | **Family** | ***df*** | **Slope** | **F Stat.** | **Adj. R^2^** | **P value** |
| --- | --- | --- | --- | --- | --- | --- |
| North | Acroporidae | 2 | -1.04 | 0.04 | -0.47 | 0.85 |
|  | Pocilloporidae | 2 | 0.99 | 2.00 | 0.25 | 0.29 |
|  | Poritidae | 2 | 2.61 | 3.58 | 0.46 | 0.20 |
|  | Faviidae | 2 | 0.07 | 0.23 | -0.35 | 0.68 |
| North-east | Acroporidae | 2 | -16.07 | 7.91 | 0.70 | 0.11 |
|  | Pocilloporidae | 2 | 2.62 | 4.99 | 0.57 | 0.16 |
|  | Poritidae | 2 | 1.17 | 1.84 | 0.22 | 0.31 |
|  | Faviidae | 2 | 0.07 | 0.23 | -0.35 | 0.68 |
| Central | Acroporidae | 2 | -1.16 | 0.04 | -0.47 | 0.86 |
|  | Pocilloporidae | 2 | 3.46 | 3.01 | 0.40 | 0.23 |
|  | Poritidae | 2 | 1.94 | 3.22 | 0.43 | 0.21 |
|  | Faviidae | 2 | 3.47 | 1.62 | 0.17 | 0.33 |
| South | Acroporidae | 2 | -21.54 | 62.04 | 0.95 | 0.02 |
|  | Pocilloporidae | 2 | 0.74 | 4.69 | 0.55 | 0.16 |
|  | Poritidae | 2 | 0.80 | 3.80 | 0.48 | 0.19 |
|  | Faviidae | 2 | 0.27 | 4.82 | 0.56 | 0.16 |
